# Case Report: A Patient with COVID-19 under Myelosuppression Induced by Chemotherapy

**DOI:** 10.4269/ajtmh.20-0678

**Published:** 2020-09-16

**Authors:** Yuki Otsuka, Taiichiro Kobayashi

**Affiliations:** Department of Infectious Diseases, Tokyo Metropolitan Cancer and Infectious Diseases Center Komagome Hospital, Tokyo, Japan

## Abstract

COVID-19 has now spread globally, and 10–20% of the cases are thought to proceed to a severe condition. However, information on COVID-19 in immunodeficient patients remains limited. We treated a 56-year-old man who developed COVID-19 after chemotherapy for mantle cell lymphoma. After 1 month of prolonged fever, the patient’s respiratory condition deteriorated rapidly, and he died. COVID-19 in immunocompromised patients after chemotherapy, even with mild symptoms, can cause rapid immune reconstitution and respiratory deterioration. Therefore, caution is advised until negative PCR test results for SARS-CoV-2 are confirmed.

## INTRODUCTION

The COVID-19 pandemic began in December 2019, and many risk factors for its onset and aggravation have now been identified. With regard to the clinical course, 10–20% of the patients are known to proceed to a severe condition after 1–2 weeks of mild symptoms.^1,2^ Previous reports have also pointed out that an immunodeficient state, such as malignancy and chemotherapy, may be a risk factor for severe COVID-19.^3–5^

However, the number of immunodeficient patients reported so far is small, and comparison of COVID-19 characteristics between immunocompetent and immunodeficient patients in terms of progress and treatment is not very clear. We treated a case of COVID-19 caused by nosocomial infection during myelosuppression after chemotherapy for mantle cell lymphoma (MCL). We report this in the present study, hoping that it will help treat immunodeficient patients in endemic areas.

## CASE PRESENTATION

A 56-year-old Japanese man presented positive status for SARS-CoV-2. The patient had a history of MCL with which he was diagnosed in September 2019. He underwent four cycles of rituximab/cyclophosphamide/vincristine sulfate/doxorubicin and hydrochloride/dexamethasone/methotrexate/cytarabine (R-hyper CVAD/MA) therapy from October 2019 to February 2020. Because of strong bone marrow suppression, R-hyper CVAD/MA therapy was discontinued, and bendamustine/rituximab (BR) therapy was commenced on March 10, 2020 (day −20). On day −4, a screening pharyngeal PCR test was performed because of a COVID-19 outbreak in another hospital, with many patients and healthcare providers in certain wards having tested positive for SARS-CoV-2. Next day, a fever of 38.0°C was recorded in this patient. On March 31 (day 1), his screening PCR test result for SARS-CoV-2 was reported positive, and he was transferred to our hospital.

**Figure 1.**
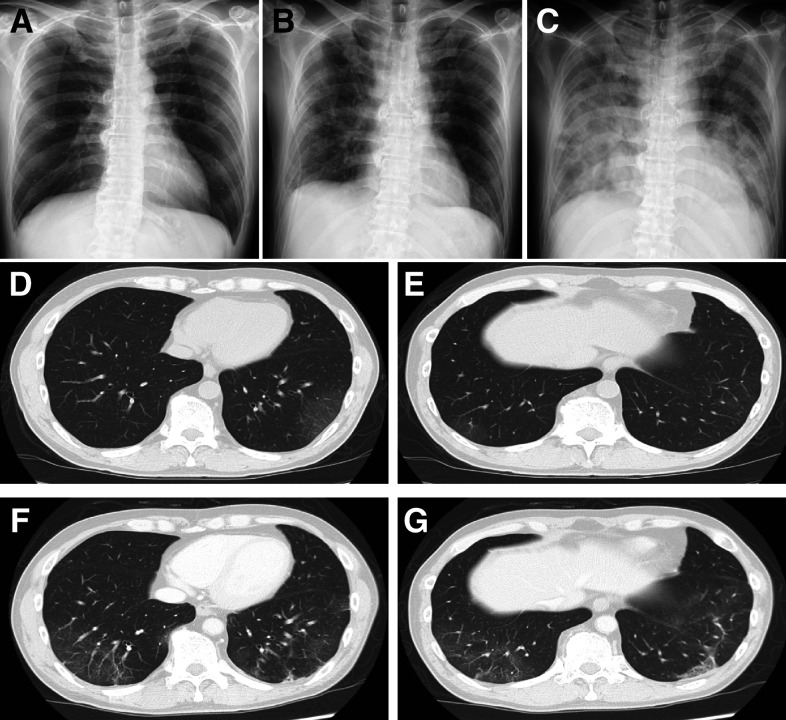
Two modalities of chest imaging studies in the COVID-19 patient. Chest X-rays. (**A**) March 31 (day 1); (**B**) April 27 (day 28); (**C**) May 4 (day 35). Chest computed tomography shows mild bilateral ground-glass opacities (GGOs) in the lower lobes (**D** and **E**: March 31, day 1). Part of the previous GGO disappeared, and a new subpleural GGO appeared (**F** and **G**: April 10, day 11).

**Figure 2.**
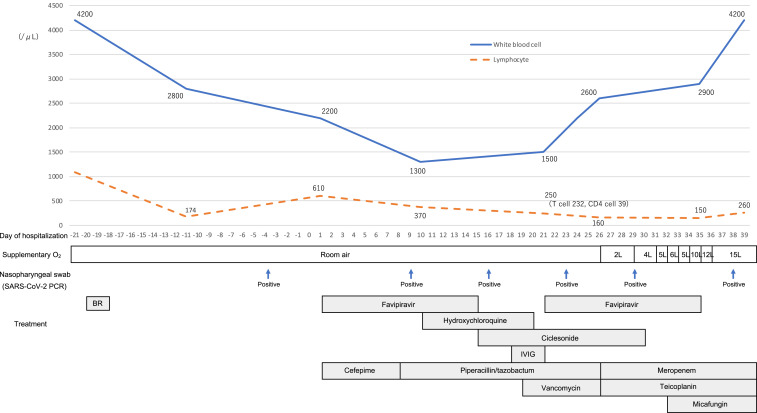
Time line of disease course and treatments from March 9, 2020 (day −21) to May 8, 2020 (day 39). Two courses of favipiravir (day 1: 1,800 mg twice daily and days 2–14: 800 mg twice daily), hydroxychloroquine (day 1: 400 mg twice daily and days 2–10: 200 mg twice daily), and inhaled ciclesonide (days 1–14: 400 μg twice daily). Polyglobin, 5 g, was administered for 3 days as intravenous immune globulin (IVIG) therapy. BR = bendamustine/rituximab.

On presentation, the patient’s vitals were as follows: 36.5°C temperature, 66/minute heart rate, 104/66 mmHg blood pressure, and 99% oxygen saturation as he breathed room air. The laboratory test reports were as follows: white blood cell (WBC) count of 2,200/μL (< 3,300), lymphocyte count of 610/μL (< 800), hemoglobin level of 11.2 g/dL (< 13.7), platelet count of 33,000/μL (< 158,000), aspartate aminotransferase level of 60 U/L (> 30), alanine aminotransferase level of 80 U/L (> 42), lactate dehydrogenase level of 237 U/L (> 222), and C-reactive protein concentration of 0.75 mg/dL (> 0.14). Renal function, PaO_2_, and PaCO_2_ were within normal limits. The chest X-ray was clear (Figure 1A), and the chest computed tomography (CT) scan showed mild bilateral ground-glass opacities in the lower lobes (Figure 1D and E). White blood cell count decreased compared with before BR therapy (Figure 2), so myelosuppression existed before COVID-19 due to chemotherapy, but pneumonia was mild as shown by the image, and the respiratory condition was stable; therefore, we determined that he had a mild symptom of COVID-19. We commenced treatment with favipiravir, and also broad-spectrum antibiotics, starting with cefepime, for probable bacterial coinfection on day 1 of admission.

From day 2, a fever greater than 38°C continued during hospitalization. Repeated PCR examinations for SARS-CoV-2 showed persistent positive results. Blood and sputum were cultured on a regular basis, but no significant bacterium grew. Computed tomography scans were reexamined to determine the cause of prolonged fever on day 11, when there was only slight improvement, with some exacerbation of pneumonia (Figure 1F and G). Because COVID-19 was considered to be the cause of fever, several available drugs that were thought to target the infection were administered (Figure 2). After that, WBC gradually increased from day 10 (Figure 2). However, from day 26, pneumonia gradually worsened, and supplementary oxygen became necessary. Blood and sputum cultures were repeatedly negative, and tests for beta-D-glucan, galactomannan antigen, cytomegalovirus antigen (C7-horse radish peroxidase), and serum cryptococcal antigen were all negative. Therefore, we determined that the only cause of pneumonia was SARS-CoV-2. Interleukin-6 increased significantly to 187 pg/mL on day 35, thus supporting the cytokine storm induced by COVID-19. Following this, oxygen requirement increased to the use of a reservoir mask at 15 L/minute on day 36; a chest X-ray showed that pneumonia had worsened sharply as oxygen requirement increased (Figure 1C). He did not wish to undergo tracheal intubation, so we could not provide mechanical respiratory support other than oxygen administration. He died on day 39.

## DISCUSSION

We treated a case of SARS-CoV-2 infection after BR therapy following R-hyper CVAD/MA therapy for MCL. Although several therapies that appeared to be effective for SARS-CoV-2 were administered, the patient’s fever lasted for 1 month, and then his respiratory condition suddenly worsened, leading to death.

Previous reports have indicated that in addition to hematological tumor types, the use of anticancer agents such as immune checkpoint inhibitors may worsen the prognosis for COVID-19.^6,7^ In this case, R-hyper CVAD/MA + BR therapy caused severe immunodeficiency, which exposed the patient to a severe SARS-CoV-2 infection. Various therapeutic agents for COVID-19 have been proposed, but their efficacy remains to be determined. Hence, the survival of COVID-19 patients depends on the patient’s own immune status, specifically the T-cell immune response. Recent articles have reported that the SARS-CoV-2 infection significantly decreases helper T-cell and regulatory T-cell counts.^8^ In this case, the WBC counts were elevated over days 21, 24, and 26, when we expected recovery from myelosuppression, but from day 26, supplementary oxygen administration became necessary for exacerbation of pneumonia. Until then, the patient’s immune system could not probably have functioned well because of myelosuppression, so the infection was manifested only in the form of fever. Over time, immune suppression may have resolved to an extent, resulting in a rapid and excessive immune response and rapid exacerbation of pneumonia. This is similar to the general clinical course of acute exacerbation of COVID-19 pneumonia. Although this occurs usually 1–2 weeks after onset in the general cases, the later occurrence here (1 month after onset) may be due to immunosuppression.^2^ This clinical course is similar to the pathology of immune reconstitution inflammatory syndrome (IRIS), wherein the immune response to pathogens becomes apparent as the immune system is restored. The main component of the COVID-19 immune response is T cells. We were unable to measure T cells frequently. Hence, this idea about IRIS is hypothetical and limited.

In this case, PCR examinations for SARS-CoV-2 persistently showed positive results. These results suggest that immunosuppressed patients prolong viral shedding. Furthermore, there are a few reports on the efficacy of corticosteroids in COVID-19.^9^ However, it has been pointed out that early use of a corticosteroid may provoke viral replication and prolong viral shedding, and therefore it is not recommended.^10–12^ Although further research results are awaited for the use of corticosteroids against COVID-19, for immunosuppressed patients, it is more important to determine the time of commencement of a treatment with a corticosteroid.

For the treatment of hematological tumors in COVID-19 highly endemic areas in the future, we believe that pre-chemotherapy PCR screening and avoidance of regimens that result in strong immunodeficiency may be required.

Most immunocompromised patients are hospitalized in Japan, so the transmission of COVID-19 in immunocompromised patients may be mainly via hospital-acquired infection. Therefore, it is critical to prevent nosocomial infections. Further assessment of cases of COVID-19 in immunosuppressed patients is awaited.

In conclusion, we can only ascertain that COVID-19 in immunosuppressed patients (such as post-chemotherapy) may show an atypical course, and caution should be exercised in these cases. In the future, we would carefully monitor PCR results and the immune status of immunosuppressed COVID-19 patients; if pneumonia worsens and supplemental oxygen is required, we would consider active commencement of therapy using not only antiviral agents (currently remdesivir) but also corticosteroids including dexamethasone.^13^
